# Dedicated near-infrared oximeter to monitor oxygenation in the superior sagittal sinus in newborn infants: a research agenda

**DOI:** 10.1117/1.JBO.27.7.074703

**Published:** 2022-03-02

**Authors:** Gorm Greisen

**Affiliations:** a Rigshospitalet, Copenhagen University Hospital, Department of Neonatology, Copenhagen, Denmark; b Copenhagen University, Institute of Clinical Medicine, Copenhagen, Denmark

**Keywords:** phantom, oximetry, newborn, accuracy

## Abstract

**Significance:**

Cerebral tissue oximetry is imprecise and confounded by an uncertain and variable arteriovenous volume ratio. Venous saturation is better grounded in physiology. The superior sagittal sinus (SSS) is relatively large and placed under the open fontanel on the top of the head in newborn infants.

**Aim:**

To enable the development of a dedicated near-infrared-spectroscopy-based cerebral oximeter with sufficient claims on accuracy to be tested for benefit of clinical use.

**Approach:**

To set up a research agenda based on the combination of dedicated, high-fidelity digital and physical phantoms.

**Results:**

A seven-step path is outlined to identify an optode geometry with high sensitivity to variation in hemoglobin-oxygen saturation in the SSS, with little confounding by changes in the optical properties of the skin and scalp or brain tissue, or in the width of the subarachnoidal space, and that is robust to variations in the placement of the optode.

**Conclusion:**

If an oximeter that is designed after exploration of digital phantoms can produce measurements in physical phantoms with good agreement with predictions, it will contribute credibility that cannot be achieved by direct gold-standard validation in newborn human infants.

## 1 Introduction

Tissue oximetry by near-infrared light applied to the head has a special opportunity when applied to newborn and small infants. Due to their thin scalp and skull, a particularly high proportion of the signal will come from the brain.^1^ Thus, this can really be called cerebral oximetry.

Furthermore, in the clinical field of neonatology, there is a great need for minimally invasive methods of oximetry.^2^ First, sampling of blood must be minimized due to the small circulating blood volume of the infants. Second, the placement of catheters in blood vessels is difficult due to their small size and risky due to their poor defenses against infection. Third, cardiopulmonary support and intensive care are often required during the transition from intra- to extrauterine life.

A large literature describes the clinical research application of cerebral oximetry to elucidate the physiology and pathophysiology of cerebral blood flow and oxygen metabolism in newborn infants. Several oximeters are approved for clinical use in this patient group and two large-scale randomized trials are currently testing the clinical benefits and harms of the combination of monitoring of cerebral oxygenation and a guideline with suggestions of what to do if the cerebral oxygenation drops below a hypoxic threshold.^3^^,^^4^

## 2 Methodological Problems

The precision of transcranial cerebral oximetry is relatively poor. If the optode is lifted off and replaced at a slightly different position on the head, the average difference in reading will be about 5%.^5^^,^^6^ This is high compared to the interindividual standard deviation of cerebral oxygenation in normal newborn infants of 8%,^7^ or compared to the requirement of an absolute root mean square accuracy of better than 3% for pulse oximetry, another optics-based clinical monitoring technology.^8^

Tissue oxygenation is not a well-defined concept. The measured value is a weighted average of the hemoglobin saturation in all blood vessels in the optical field. Since driving pressures on the venous side of the circulation is much lower than those on the arterial side, veins need to be larger than arteries and the arterial-to-venous ratio has been estimated to 1:2^9^^,^^10^ and to 1:3 and variable.^11^

This leads to three problems. First, the question of accuracy is not simple, since there is no physiological reference comparable to that of pulse oximetry, where co-oximetry on arterial blood can be considered the gold standard. Calibration and validation of cerebral oximeters therefore typically use desaturation studies in healthy adult volunteers and are based on an assumed, fixed arterial-to-venous ratio.^12^ Perhaps this is one reason why different devices give widely different values in dynamic blood lipid phantoms^13^ and during spontaneous deoxygenation in preterm infants.^14^ Second, if the arterial-to-venous ratio changes during the clinical course, this may be misinterpreted as a change in cerebral oxygenation.^11^ Third, it reduces the sensitivity of tissue oxygenation to decrease in cerebrovenous oxygenation due low blood flow, e.g., due to hyperventilation, or due to increased oxygen needs, e.g., during seizures, due to “dilution” of the signal with arterial blood of normal oxygenation.

Several attempts have been made to measure cerebrovenous oxygen saturation in newborn infants by near-infrared spectroscopy. Tilting the head down or occlusion of the jugular veins on the neck for a few seconds to induce pooling of blood in the cerebral veins has been used to measure the relative increases in oxy- and deoxyhemoglobin by continuous wave spectroscopy.^15^ Average values in healthy term newborn infants were 65%,^16^ close to the average normal values in healthy adults. These methods, however, do involve manipulation of potentially sick and vulnerable newborn infants and are not always successful. Analysis of signal changes induced by positive pressure ventilation is also possible and has given similar values^17^ but has not been explored further, and the analysis of signal changes induced by spontaneous ventilation is often not possible.^18^

## 3 Opportunity

**Fig. 1:**
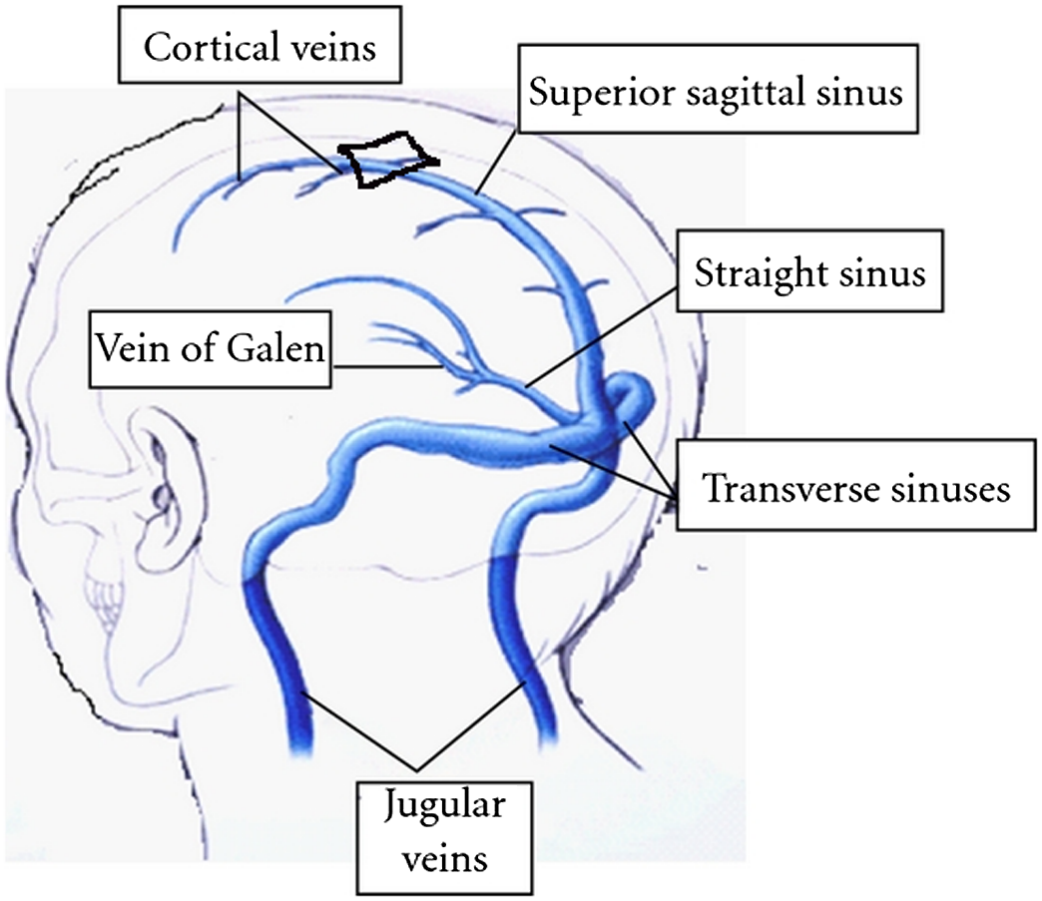
The large cerebral veins and sinuses. The sinuses carry venous blood. The sinuses are formed by dura, the fibrous sheath that covers bone, and the SSS is located just below the bone at the top of the skull. It drains all venous blood from the upper parts of the brain hemispheres, whereas the straight sinus drains blood from the basal parts of the hemispheres. The blood flows from front to back of the head and via the transverse sinuses to the internal jugular vein. Unfortunately, the internal jugular vein also drains some extracranial tissues, so blood sampled there is not a perfect measure of cerebrovenous oxygen saturation.^19^ The approximate location of the anterior fontanel is indicated by the black diamond (modified from P.K. Sasidharan, CC BY 3.0, via Wikimedia Commons).

**Fig. 2:**
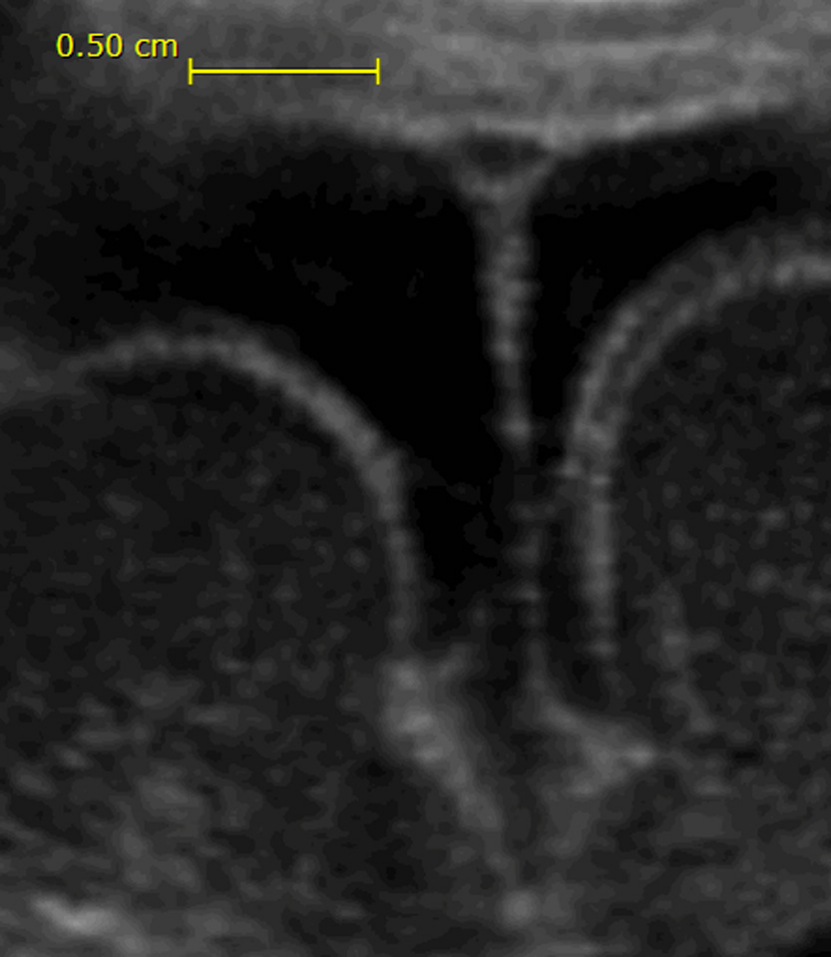
Cross-sectional image of the SSS in a 27-week gestation preterm infant at the level of the anterior fontanel. Although ultrasound can penetrate bone, good images require the fontanel as an acoustic window. The black layer between the inner surface of the skull/fontanel and the surface of the brain is unusually wide in this infant, about 7 mm, and is constituted by cerebrospinal fluid in the subarachnoidal space. The subarachnoidal space separates the two brain hemispheres in a deep V-shape. The thin line in the middle represents the falc cerebri. On top of this, the triangular SSS is seen, measuring 2 to 3 mm on each side. It is black on the ultrasound image, since flowing blood, such as cerebrospinal fluid, gives few echoes to ultrasound.

In newborn and small infants, the bones of the skull are not yet fused, and the anterior fontanelle on the top of the head is patent for the first year of life. Just below the anterior fontanelle, the two layers of falx cerebri (the fibrous sheath that separates the two brain hemispheres) split into two before it fuses with the periosteum (the fibrous sheath that covers the inside of the bone). The triangular space constitutes a sinus that is filled with venous blood flowing from the superior parts of the hemispheres (Figs. 1 and 2). This sinus can be interrogated at the level of the anterior fontanel, thus avoiding interposing bone and red bone marrow, or more posteriorly through bone, as is usually done for cerebral oximetry.

The short distances, the large amount of venous blood, and the well-defined anatomy should make it possible to obtain a nearly purely venous oxygenation and thereby to circumvent the three problems delineated above. This approach, however, has not yet been tried.

## 4 Limitations of *In-Vivo* Calibration and Validation

It is not ethically permissible to sample blood from the superior sagittal sinus (SSS) in newborn infants although this was routinely done at the outset of modern neonatology. Even cannulation of the internal jugular vein cannot be done for a research purpose, and catheterization of veins on the neck is rarely done for clinical purposes in newborn infants, so opportunistic blood sampling cannot be planned on a large enough scale to obtain statistically firm estimates of accuracy.

The brains of experimental animals, such as piglet or lambs, are considerably smaller than the brain of human infants, and although the anatomy of the SSS is similar, the differences in size will subtract significantly from the value of validation in these animals.

## 5 Definitive Role for Phantoms

High-resolution imaging is possible by ultrasound as well as MR, therefore precise three-dimensional anatomy can be defined and used for building physical phantoms as well as *in-silico* models of photon propagation (digital phantoms). Only short source–detector distances need to be examined, so the overall size of the phantoms can be small. Comparison of results from physical and digital phantoms and exploring and possibly resolving any significant differences may enhance the trust in the validity of the results.

## 6 Challenge of Anatomical Variability

One challenge for the translation of results from phantom work to the application of an instrument on patients or research subjects is the anatomical variability. A digital phantom study of the reliability of frequency-domain NIRS across the age span from birth to adult age used MR images to model the “superficial layer” (i.e., skin-scalp-bone + subarachnoidal space), subarachnoidal space, and brain tissue.^1^ Compared with children below 1 year of age, with a thin scalp and skull, a very attenuated response to changes in the absorption coefficient ($\mu_{a}$) in brain tissue was demonstrated in adults and larger children. By chance, perhaps, one of the MR images of a young child used for this study displayed an open sylvian fissure (a space of subarachnoidal fluid in the parietotemporal region), just under the row of detectors. The Monte-Carlo simulations demonstrated how this space attenuated the sensitivity to changes of $\mu_{a}$ in brain tissue.

The variability of the development of gyration of the brain and closure of the Sylvian fissure is well known. Although there is less variation at the top of the hemispheres, near the SSS, the width of the subarachnoidal space does differ among infants.^20^ The width increases with growth and development of preterm infants, and furthermore, infants born very preterm when 4 to 8 weeks old have wider subarachnoidal space compared to infants with the same biological age, but born more recently. The average width in the first scan was 1.5 mm, with an upper limit of the “normal range” of 4 mm, while in the latest follow-up scans the mean value was 3 mm and the upper limit of the normal range was 6 mm. The head circumference ranged from 220 to 340 mm.

Similarly, the thickness of skin, bone, and the subarachnoidal space has been studied over the temporoparietal region. The thickness of skin varied from 1 to 3 mm and bone from 1 to 5 mm.^21^ Digital phantom work has previously demonstrated that superficial layers with a thickness up to 6 mm do not significantly affect the measurement of brain oxygenation.^22^

## 7 Phantom Research Agenda

An agenda for developing a device to measure oxygen saturation in the SSS in newborn and small infants could include the following.

A. A series of “digital phantoms” built to represent the typical anatomy, as well as the variation in the relative size of the compartments, and the growth in size with age. The subarachnoidal space and the SSS could be represented as separate volumes, whereas the hemoglobin in the smaller blood vessels in the superficial layer (skin-scalp-bone), the subarachnoidal space, the brain, and potentially the red bone marrow of the skull could be included in the optical properties in the respective compartments. Phantoms representing a range of likely optical properties must be explored.
B. Source–detector geometries that are parallel with as well as perpendicular to the SSS are explored in the digital phantom, as regards their sensitivity to changes in the $\mu_{a}$ in the SSS as compared to the sensitivity to changes in the $\mu_{a}$ in the other compartments.
C. The robustness to variation in the optode position is determined.
D. A sensor geometry is chosen that achieves the optimal balance of sensitivity to changes in $\mu_{a}$ in the SSS, minimal bias from changes in $\mu_{a}$ or scattering coefficients in the superficial and brain compartments, and robustness to deplacement of the optode.
E. A series of physical phantoms of the same compartments are built to represent the same variability in proportions and size. The SSS could be connected to a pump that circulates blood that can be manipulated as regards its oxygen saturation. The brain compartment is constructed so it can function as a blood–lipid phantom, e.g., using yeast and bubbling of oxygen to vary the oxygen saturation. The superficial layer can be represented by a slab of solid material with known optical properties. A similar approach has previously been used to examine the possibility of estimating the saturation of the blood in the transverse sinus in adults.^23^
F. A prototype oximeter built to the specification of the optimal optode geometry, using the best practice choice of wavelengths and algorithms.
G. Verification of the performance of the oximeter on the physical phantoms using changes in oxygen saturation in the SSS and the brain compartments, respectively, as well as variation in optode placement.

## 8 Next Steps

Gold-standard verification in human infants is impossible, but alternative *in-vivo* methods are available. One option is to compare with the results of the methods based on brief obstruction of venous outflow from the brain, as described above. Many years ago, two different methods of measuring cerebral blood volume by continuous wave near-infrared spectroscopy revealed significant differences.^24^

Another way is to examine the “construct validity,” i.e., to compare measured responses to expected responses to stimuli for which there already is good evidence for the magnitude. For instance, the validity can be examined by comparing the absolute changes between this measure of cerebrovenous saturation (in %) and arterial blood saturation (in %) as monitored by pulse oximetry during spontaneous variations in oxygenation, which are common in preterm infant due to periodic breathing or frank apnea.^25^ When arterial oxygen saturation stays within the normal range, compensatory changes in cerebral blood flow or cerebral metabolism of oxygen are not expected^26^ and arterial and venous saturation is expected to occur in parallel. This has been used to measure cerebral blood volume and flow in newborn infants using “oxygen as a tracer.”^15^ Also, robustness against changes in venous pressure can be examined. Spontaneous changes in intrathoracic pressures happen during ventilatory support, and these are expected to cause passive dilatation of cerebral veins, but not to affect cerebrovenous saturation.

These comparisons, however, will all be far from ideal, and thus the validation obtained by phantom studies will be of great value, and can potentially contribute significantly to the trust in a venous oximeter.

## 9 Conclusion

Good agreement between the performance of an “SSS oximeter” as predicted from digital phantom evidence and actual performance on a series of physical phantoms with realistic variations in anatomic features and in optical properties could contribute significantly to the credibility of the accuracy of such an oximeter.
